# Hyponatremia-Induced Seizure in a Patient With Psychogenic Polydipsia: A Case Report

**DOI:** 10.7759/cureus.37710

**Published:** 2023-04-17

**Authors:** Ammar Y Bafarat, Suhail A Labban, Nada Alhatmi, Hanan Aly, Dalal M Bashah, Fatma Alshaiki

**Affiliations:** 1 General Practice, King Abdulaziz Hospital, Jeddah, SAU; 2 Medicine, King Saud Bin Abdulaziz University for Health Sciences College of Medicine, Jeddah, SAU; 3 Psychiatry and Behavioral Sciences, King Abdulaziz Hospital, Jeddah, SAU; 4 Psychiatry and Behavioral Sciences, Sohag University, Sohag, EGY; 5 Internal Medicine, King Abdulaziz Hospital, Jeddah, SAU; 6 Internal Medicine, Saudi Ministry of Health, Jeddah, SAU

**Keywords:** bullying, anorexia nervosa (an), delusional disorder, hyponatremia, psychogenic polydipsia

## Abstract

Psychogenic polydipsia is a rare condition characterized by overconsumption of water. It can lead to water intoxication, which is potentially a life-threatening situation. Moreover, it usually occurs in patients with mental disorders, mainly schizophrenia. This report discusses a successful treatment of a 16-year-old male with psychogenic polydipsia and delusional disorder presenting to the emergency room with a hyponatremia-induced seizure. After stabilizing the patient, he was referred to a psychologist, and behavioral therapy was conducted. Post-discharge follow-ups revealed that behavioral therapy and the use of self-monitoring technique were effective in controlling the patient's condition. His water intake was reduced from 15 liters per day to three liters per day. This case highlights the importance of psychological assessment for patients with features suggestive of psychogenic polydipsia. It also highlights the need for immediate admission and prompt treatment for such patients as it is a high-risk condition.

## Introduction

Polydipsia is a clinical disorder characterized by excessive water intake. It can show up as a symptom of multiple conditions, such as diabetes insipidus, diabetes mellitus, Addisonian crisis, Conn's syndrome, and chronic renal failure. However, psychogenic polydipsia (PPD), also known as compulsive water drinking or self-induced water intoxication, is often observed in patients with mental disorders, such as schizophrenia, bipolar disorder, and depression with psychotic features [1]. It is also observed in patients with anorexia nervosa as a strategy to reduce hunger [2]. It is a serious condition that occurs in 6% to 20% of psychiatric patients and requires close monitoring [3]. 

Hyponatremia (sodium <135 mmol/L) is shown in 10% to 20% of patients presenting with compulsive water drinking. Left untreated, it can progress to water intoxication (a severe form of hyponatremia) and cause seizures, cerebral edema, and death [3]. Such presentation in patients with delusional disorder is very rare in the literature. Therefore, In this report, we present a case of hyponatremia-induced seizure secondary to psychogenic polydipsia in a patient with a newly diagnosed delusional disorder.

## Case presentation

A 16-year-old male patient, not known to have any chronic medical illnesses, presented to the emergency room (ER) with a history of tonic-clonic seizure six hours prior to the presentation. The seizure lasted for two minutes with a loss of consciousness, uprolling of the eye, and biting of the tongue. The patient woke up after five minutes without any intervention. After regaining consciousness, he vomited and had a headache associated with photophobia. He was admitted to the intensive care unit (ICU) as a case of severe hyponatremia.

A detailed collateral history was taken from the father. It revealed a history of weight loss (23 kg over one year) as the patient has been following a strict keto diet and intermittent fasting. Since starting the diet, the patient started drinking approximately 15 liters of water daily with a preference for cold water. When the family tried to restrict his water consumption, he drank water from the sink. The father denied any history of previous seizures. However, there was a history of untreated anorexia nervosa and a previous visit to another hospital due to malnutrition. The patient's sodium level was 121 mmol/l at that time, and he was discharged on multivitamins after normalization of his sodium level. The patient's current main complaints were excessive thirst and frequent urination. He denied any other symptoms. Moreover, he was not on any medications and did not have any allergies. There was no history of recent travel or contact with a sick patient. He comes second among five brothers and lives with his parents. He is in eleventh grade, and he is at the top of his class.

The patient's serum sodium level was 112 mmol/l (severe hyponatremia) upon admission. His vital signs were within normal limits. He was conscious, alert, oriented, and breathing well at room temperature, but he looked drowsy and ill. Physical examination was unremarkable. His management consisted of free water restriction, 100 ml intravenous infusion of 3% hypertonic saline over 30 minutes, diazepam 5 mg, and intravenous infusion of normal saline 500 ml in one hour and maintenance at 63 cc/h. All the necessary investigations were ordered, and the patient's vital signs were closely monitored. The target serum sodium level was not more than 118 mmol/l in six hours to avoid overcorrection symptoms. Instructions to hold an intravenous infusion of normal saline were given in that case. The syndrome of inappropriate secretion of antidiuretic hormone (SIADH) was ruled out by the endocrinology team, according to the patient's urine analysis. Adrenal insufficiency was also ruled out, as his morning cortisol level was normal. Other investigations ruled out all medical conditions that are known to be etiologically related to hyponatremia. A provisional diagnosis of severe hyponatremia induced by psychogenic polydipsia was made as he had low urine and serum osmolality. Furthermore, a psychiatric consultation was requested. A nutritional assessment revealed that the patient was underweight (BMI of 17.6 kg/m^2^). His nutritional requirements were calculated. The impression and plan were explained in detail to his father.

Two days later, the patient's sodium level was corrected (132 mmol/l), and intravenous fluid was stopped. He had no complaints and was transferred to a normal bed. In the following two days, the patient's serum sodium level remained within normal limits. He was vitally stable, conscious, alert, and oriented. The patient was planned for discharge after a psychiatric evaluation. A summary of laboratory investigations is provided in Table 1.

**Table 1:** Summary of laboratory values BUN - blood urea nitrogen

	Variable	Result	Normal range
Upon admission	Serum sodium	112 mmol/l	136-145 mmol/l
	Serum urea	2 mmol/l	2.5-6.4 mmol/l
	BUN	5.61 mg/dl	6-20 my/dl
	Serum osmolality	232.2 mOsm/kg	275-295 mOsm/kg
	Urine sodium	12.29 mmol/l	20-40 mmol/l
	Urine osmolality	40 mOsm/kg	500-800 mOsm/kg
	Glucose	111 mg/dl	90-130 mg/dl
	Morning cortisol	17.80 ug/dl	6.7-22.6 ug/dl
Before discharge	Serum sodium	139 mmol/l	136-145 mmol/l
	Serum urea	6.8 mmol/l	2.5-6.4 mmol/l
	BUN	19.07 mg/dl	6-20 my/dl
	Serum osmolality	290.4 mOsm/kg	275-295 mOsm/kg
	Glucose	100 mg/dl	90-130 mg/dl

After stabilizing the patient, a full psychiatric assessment was performed. He reported excessive thirst, lethargy, and numbness in his extremities when not drinking water every hour. He also reported a firm, unshakable belief that he would die if he did not drink water every hour. In addition, he said that he consumes the highest amount of water upon waking up since he has not drank any water for several hours during his sleep. There were no signs or symptoms suggestive of depression, anxiety, or psychosis. Moreover, he did not meet the criteria for body dysmorphic disorder (BDD), as he is content with his current weight. However, he expressed dissatisfaction with his weight and appearance before losing weight. A provisional diagnosis of psychogenic polydipsia and delusional disorder (DD) was made, and the differential of obsessive-compulsive disorder (OCD) was considered. The patient was then scheduled for a follow-up in the outpatient clinic in two weeks. A psychotherapy appointment was also booked. Impression and plan were explained in detail to the father.

During the follow-up with a psychiatrist and a psychotherapist two weeks later, the patient reported that he started the diet with the intention of losing weight because his colleagues in school bullied him. During this time, the patient developed an irresistible urge to drink large amounts of water, which was observed by his family. This urge is due to his firm, unshakable belief that he would die if he did not drink this much water. The amount of water consumption increased remarkably with time, which led him to eventually develop hyponatremia and a seizure. A cognitive behavioral therapy session was performed, and the technique of self-monitoring was used, in which the patient was instructed to monitor his water intake and limit it to three liters per day. Additionally, he was given a log diary of water intake to divide the three liters throughout the day. He was also provided with information regarding the dangerous health consequences of excessive water consumption. Furthermore, his parents were instructed to reward him when he sticks to drinking only three liters of water per day. There were no other active psychiatric issues. The patient and his family were calm, cooperative, and willing to follow the instructions.

A follow-up six months later revealed that the patient's urge to drink water was controlled. He followed the instructions and drank only three liters per day, according to the father. He also denied any fear of death or somatic symptoms with the current water consumption plan (2-3 L/day). His current BMI is 25.

## Discussion

Psychogenic polydipsia can potentially be fatal. There are multiple reports of life-threatening consequences, such as seizures, rhabdomyolysis, cerebral edema, aspiration pneumonia, and crural compartment syndrome as a result of water intoxication [4]. Water intoxication is thought to be explained by three factors: polydipsia, which is defined as the water consumption of more than three liters per day and up to 10 or 15 liters in extreme cases, inability to excrete water due to kidney disturbance or inappropriate secretion of antidiuretic hormone, and sensitivity of the brain to hyponatremia [5]. 

In this case, bullying at school was thought to be a potential trigger for the development of anorexia nervosa and psychogenic polydipsia. The patient was subjected to bullying about his weight by his colleagues, which eventually led him to follow a strict diet and consume large amounts of water. This is consistent with studies that identified bullying as a risk factor for a wide range of mental disorders. In one study, being exposed to bullying and critique of appearance was found to be the most common experience among patients with body dysmorphic disorder (BDD) [6]. In another study, 36% to 59% of patients with eating disorders (ED) reported having been bullied about their appearance at some point. Moreover, compared to healthy individuals, those with EDs were twofold to threefold significantly more likely to have been bullied about their appearance prior to the onset of their ED [7].

There are various methods for the treatment of psychogenic polydipsia in the literature. The gold standard is fluid restriction, which is an inexpensive form of treatment that has shown great effects in inpatient settings [3]. The use of medications, such as demeclocycline, propranolol, and captopril, has shown inconsistent results. However, the use of atypical antipsychotics such as clozapine has shown success in alleviating the symptoms of psychogenic polydipsia in three cases, but it remains unproven in large trials [3]. The use of low-dose risperidone and olanzapine has improved polydipsia in another case report [8].

Apart from pharmacological therapy, cognitive behavioral therapy remains the mainstay of management for psychogenic polydipsia [3]. In this report, the patient was successfully treated with cognitive behavioral therapy and the design of a log-diary of water intake. The technique of self-monitoring was also used. Another report showed that the patient's water consumption was significantly reduced by using two ways. Firstly, she was allowed to drink water only through a narrow straw which made consumption more difficult, and she was asked to do a low-preference activity or task. Secondly, water refusal was positively reinforced with edible rewards, such as candy and gum, and a period of no required activity or task. This method of reinforcing the desired behavior and punishing non-desired behavior was proven to be successful as well [9]. Other methods of behavioral therapy in the literature include stimulus control, response prevention and differential reinforcement, and relaxation training to reduce feelings of distress that accompany the urge to drink water [10].

## Conclusions

Hyponatremia caused by psychogenic polydipsia is a serious condition that can put patients in life-threatening situations. Therefore, it is crucial to immediately admit and closely monitor patients presenting with severe hyponatremia. Additionally, psychological assessment for such patients is of immense value, as psychogenic polydipsia is associated with mental disorders. Behavioral therapy and the technique of self-monitoring by using a log diary were proven to be effective in this case. More clinical trials are needed to formulate a guideline for treating this condition.
